# Expression of Phosphoinositide-Specific Phospholipase C Isoforms in Native Endothelial Cells

**DOI:** 10.1371/journal.pone.0123769

**Published:** 2015-04-13

**Authors:** Delphine M. Béziau, Fanny Toussaint, Alexandre Blanchette, Nour R. Dayeh, Chimène Charbel, Jean-Claude Tardif, Jocelyn Dupuis, Jonathan Ledoux

**Affiliations:** 1 Research Center, Montreal Heart Institute, Montreal, Qc, Canada; 2 Department of Molecular and Integrative Physiology, Université de Montréal, Montreal, Qc, Canada; 3 Department of Pharmacology, Université de Montréal, Montreal, Qc, Canada; 4 Department of Medicine, Université de Montréal, Montreal, Qc, Canada; Penn State Hershey College of Medicine, UNITED STATES

## Abstract

Phospholipase C (PLC) comprises a superfamily of enzymes that play a key role in a wide array of intracellular signalling pathways, including protein kinase C and intracellular calcium. Thirteen different mammalian PLC isoforms have been identified and classified into 6 families (PLC-β, γ, δ, ε, ζ and η) based on their biochemical properties. Although the expression of PLC isoforms is tissue-specific, concomitant expression of different PLC has been reported, suggesting that PLC family is involved in multiple cellular functions. Despite their critical role, the PLC isoforms expressed in native endothelial cells (ECs) remains undetermined. A conventional PCR approach was initially used to elucidate the mRNA expression pattern of PLC isoforms in 3 distinct murine vascular beds: mesenteric (MA), pulmonary (PA) and middle cerebral arteries (MCA). mRNA encoding for most PLC isoforms was detected in MA, MCA and PA with the exception of η2 and β2 (only expressed in PA), δ4 (only expressed in MCA), η1 (expressed in all but MA) and ζ (not detected in any vascular beds tested). The endothelial-specific PLC expression was then sought in freshly isolated ECs. Interestingly, the PLC expression profile appears to differ across the investigated arterial beds. While mRNA for 8 of the 13 PLC isoforms was detected in ECs from MA, two additional PLC isoforms were detected in ECs from PA and MCA. Co-expression of multiple PLC isoforms in ECs suggests an elaborate network of signalling pathways: PLC isoforms may contribute to the complexity or diversity of signalling by their selective localization in cellular microdomains. However in situ immunofluorescence revealed a homogeneous distribution for all PLC isoforms probed (β3, γ2 and δ1) in intact endothelium. Although PLC isoforms play a crucial role in endothelial signal transduction, subcellular localization alone does not appear to be sufficient to determine the role of PLC in the signalling microdomains found in the native endothelium.

## Introduction

Vascular endothelium, a thin-cell monolayer lining blood vessels walls, is ideally positioned to detect and transduce biochemical and physical information. Endothelial cells (ECs) are essential regulators of vascular tone, cellular adhesion and vascular smooth muscle cell (SMC) proliferation. The wide variety of stimuli sensed by ECs necessitates an intricate web of intracellular signalling components, including phosphoinositide-signalling involving phospholipases C (PLC) as main players.

PLC are calcium (Ca^2+^)-dependent phosphodiesterases that hydrolyze phosphatidyl-inositol bisphosphate (PIP_2_) into 1,2-diacylglycerol (DAG) plus inositol trisphosphate (IP_3_) [1,2]. Once generated, IP_3_ quickly diffuses in the cytoplasm and activates IP_3_ receptors (IP_3_R) located on the endoplasmic reticulum (ER) membrane, evoking a rapid release of Ca^2+^ into the cytoplasm. PLC can also regulate several cellular functions through DAG production, via activation of protein kinase C [3] and through modulation of PIP_2_ levels [4]. To date, a total of 13 mammalian PLC isoforms have been identified and classified into 6 families: β(1–4), γ(1–2), δ(1,3,4), ε, ζ and η(1–2) [5]. The PLC families possess distinct mechanisms of activation. For example, PLCβ isoforms are activated by Gα and Gβ/γ subunits of heterotrimeric G proteins whereas PLCγ isoforms are activated by tyrosine kinase receptors and PLCε can be activated by both [5]. PLC isoforms can also be distinguished by their molecular weights, Ca^2+^-sensitivity and subcellular localization: together, these suggest that each isoform might have a specific role in the modulation of physiological responses and this is further supported by several studies using knockout animal models [5–8]. For example, PLCε-null mice display abnormal development of the aortic and pulmonary valves [9]. The absence of PLCβ2 expression leads to decreased intracellular calcium release and superoxide production in neutrophils [10]. These studies strengthened the notion that each PLC isoform has a specific and complementary role in physiology.

Downstream of PLC activation, IP_3_R-dependent Ca^2+^ release is a key player in endothelial function. Indeed, these endothelial intracellular Ca^2+^ dynamics play a major role in the generation of vasoregulatory signals [11–14]. Although highly dynamic, endothelial Ca^2+^ is finely tuned, as expected for a process with a significant functional impact. An increasing body of evidence suggests that spatially restricted Ca^2+^ signals are essential regulators of endothelial function. Of the endothelial local Ca^2+^ signals characterized, Ca^2+^ pulsars are spontaneous IP_3_R-dependent Ca^2+^ release events that occur within the myoendothelial projection (MEP) [11]. Although our current knowledge regarding the regulation of spatially-localized Ca^2+^ signalling in the endothelium is limited, the involvement of IP_3_/IP_3_Rs indicate a role for PLCs in the control of Ca^2+^ signals such as pulsars. A better understanding of the expression and subcellular localization of PLCs in native ECs is necessary.

Cell-specific expression of individual PLCs is believed to be involved in modulating particular functions. Roles for PLC β3 [15], γ1 [16] and δ [17] have been reported in ECs and the expression of PLC isoforms was recently examined in human umbilical vein endothelial cells (HUVECs) [18]. However, placing ECs in culture results in phenotypic changes [19], including the loss of MEP and Ca^2+^ pulsars, suggesting that the regulatory pathways might be altered as well. Hence, the expression profile of PLCs and the associated signalling pathways may differ between HUVECs and native ECs. Endothelium is a specialized tissue whose functions vary depending on the vascular bed, suggesting that PLC expression might also vary from one vascular bed to the other [20–22]. Therefore, it is crucial to determine the expression and subcellular distribution of PLC isoforms in native ECs from different vascular beds. In this study, we determined the expression of PLC isoforms in murine ECs freshly isolated from mesenteric (MAECs), pulmonary (PAECs) and middle cerebral arteries (MCAECs).

## Materials and Methods

### Tissue preparation

All animal manipulations were performed in accordance with the Canadian Council on Animal Care guidelines. The Montreal Heart Institute Animal Research Ethics Committee approved all animal studies (Protocol number: 2011-33-01). Resistance arteries: (3^rd^ or 4^th^ order MA, PA and MCA) were isolated from 3–4 months old C57BL/6 mice. Prior to experimentation, arteries were carefully cleaned of adipose and connective tissue in cooled HEPES solution.

### Molecular biology

Total RNA from MA, PA and MCA was extracted using RNeasy Micro kits (Qiagen). As control, total RNA was isolated from brain and testis using RNeasy Lipid tissue Mini Kit (Qiagen) and from blood using QIAmp RNA blood Mini kit (Qiagen). The purity and concentration of RNA were assessed using a Nanodrop spectrophotometer. Next, reverse transcription was performed using iScript kits (Bio-Rad) according to manufacturer’s instructions. Primers (Table 1) and 5 ng of cDNA were used for PCR amplification in a final volume of 25 μl. Following denaturation at 94°C, 45 cycles of DNA amplification were performed using Taq DNA polymerase (Invitrogen) at 94°C for 45 sec, 55°C for 30 sec and 72°C for 90 sec. A final extension step was performed for 10 min at 72°C. Electrophoresis of amplicons on 3% ethidium bromide stained agarose gels was carried out. PCR product sizes were estimated using the Gene ruler low range DNA ladder (Thermoscientific) and compared to theoretical amplicon size (n≥3, Table 1).

**Table 1:** List of primer couples for PLC isoforms.

Gene	Forward (5’ → 3’)	Reverse (5’ → 3’)	Amplicon size
PLCβ1	CTGAGCGGAGAAGAAAATGG	ACACAGCGACATCCAGACAG	185
PLCβ2	TCAACCCTGTTCTATTGCCCC	CGGAGGATAACAGGAGAGGC	240
PLCβ3	AACTAGCCGCTCTCATTGGG	ACTGAGGGAGGAGCTAGTGG	176
PLCβ4	GGAAGTGCCCTCTTTCTTGC	GCCTTCACTCTTCCACGTCA	130
PLCγ1	AGATCCGTGAAGTTGCCCAG	TCAGCCTTGGTTTCCGGAAA	193
PLCγ2	GATCATGGAGACTCGGCAGG	GACAAACTGGGTGCCGTAGA	181
PLCδ1	GCAAGATCATCGACCGCTC	CGTAGCTGTCATCCACCTGT	165
PLCδ3	GACAGCAGCACCAAAAGGC	CGTGAGCGGATCTTGAGGAG	113
	CAGCAACTGACCCGAGTGTA	CTCAGGGTCAAAGGTGGTGT	222
PLCδ4	TTCAATCCTGAGAGGCCAAT	TCCACTTTGGGGAGTTGTTG	86
PLCε	GGAGCCAACGTCTGTCTGAA	GAGTTTGGGAGCTGTGTGGA	118
PLCζ	AGACTTCCTGCTTTCGGACA	TGTCGGTTCCTATCCTCTCG	137
PLCη1	CCGCAGAAAAGTCAGGCAAA	AGCTCCTCCACAGTCAGGT	171
	AGTAGGGCAGTGGGTTGAAG	TACACAAACTCCGTGGCAGC	199
PLCη2	CGGCAGAGGGTGAAACAGAT	CTCGGCGGGTAGACATCATC	109
	TGTTCATGTGGCTGTCAGTG	GACTTGGCTTCTGGCTTTTG	194

For quantitative real-time PCR (qPCR), ECs were freshly isolated from MA, PA and MCA as previously described by Socha et al [23]. Cell dissociation was performed in a tube containing 137 mM NaCl, 5.6 mM KCl, 1 mM MgCl_2_, 10mM HEPES, 10 mM glucose, 2 mM CaCl_2_, 1.0 mg/ml DTT, 0.1% BSA and with enzymes 0.62mg/ml (MAECs) or 0.31mg/ml (PAECs and MCAECs) papain (Sigma, P4762), 1.5mg/ml (MAECs) or 0.75mg/ml (PAECs and MCAECs) collagenase (Sigma, C8051). Arterial segments were incubated in digestion solution for 25 min at 37°C followed by a triple wash in enzyme-free solution and then gently triturated. SMC-deprived EC tubes were visually identified with a Zeiss Axiovert A1 microscope and individually collected with patch micropipettes to ensure the purity of the isolated ECs. ECs mRNA was extracted and purified (RNeasy Plus micro kit, Qiagen) before amplification using MessageBOOSTER Whole Transcriptome cDNA synthesis kit for qPCR (Epicentre Biotechnologies). Reverse transcription was performed using iScript kit (Bio-Rad) according to the manufacturer’s instructions. The purity of the isolated ECs was further assessed by qPCR analysis of the relative abundance of endothelial specific (CD31) and SMCs specific (SM22) mRNA (CD31/SM22 ratio) in MAECs, PAECs and MCAECs (S1 Fig). qPCR was performed using a Stratagene MX3005 system using iTaq fast Syber Green with ROX (Bio-Rad) and are normalized to cyclophilin A expression.

### Immunocytochemistry

MA were cut longitudinally, and pinned (endothelium *en face)* on a Sylgard block. Arteries were fixed with 4% paraformaldehyde, permeabilized with 0.2% Triton X-100 then blocked with 4% normal donkey serum. Following overnight incubation at 4°C with primary antibodies (1:500; PLCβ3 Abcam #ab52199; PLCδ1 Abcam #ab154610; PLCγ2 Abcam #ab18983) in 0.1% Triton X-100, arteries were incubated for 1h with an Alexa Fluor 555-conjugated donkey anti-rabbit secondary antibody (Invitrogen). DAPI was applied to stain nuclei prior mounting for confocal imaging. Autofluorescence of the internal elastic lamina was also acquired. Fluorescence emissions were detected using a Zeiss LSM 510 confocal microscope (63X oil objective/1.4, ex: 405 nm, 488 nm and 543 nm). All images were deconvolved with Huygens professional software using experimentally determined point spread functions (PSFs) and reconstructed with Zen 2009 Light Edition.

### Western blotting

Protein extraction from MA was carried out by tissue immersion in ice-cooled acetone, 10% trichloroacetic acid and 10 mM dithiothreitol (DTT). Tissue was then lyophilized, disrupted and heated at 95°C for 10 minutes in SDS-gel sample buffer (60 mM Tris-HCl, pH 6.8, 2% SDS, 10% glycerol, 0.01% bromophenol blue, 0.1 M DTT) [24,25]. Finally, proteins were extracted by continuously mixing samples in SDS-gel sample buffer overnight at 4°C. Proteins were separated by SDS-PAGE on 7.5% acrylamide gels and transferred to nitrocellulose membranes (Bio-Rad). Membranes were blocked using 5% fat-free dry milk and then incubated with primary antibodies (1:1000; PLCβ3 Abcam #ab52199; PLCδ1 Abcam #ab154610; PLCγ2 Abcam #ab18983) for 1 hour at room temperature. Membranes were then washed and incubated with HRP-conjugated goat anti-rabbit secondary antibody (Jackson ImmunoResearch) for 1 hour at room temperature. Immunoreactive bands were revealed by enhanced chemiluminescence (Western Lightning Plus, PerkinElmer).

### Statistical analysis

Data are presented as means ± SEM. One-way ANOVA with Turkey’s multiple comparisons test was used to compare the means; P<0.05 was considered significant.

## Results

### mRNA expression of PLC isoforms

**Fig 1:**
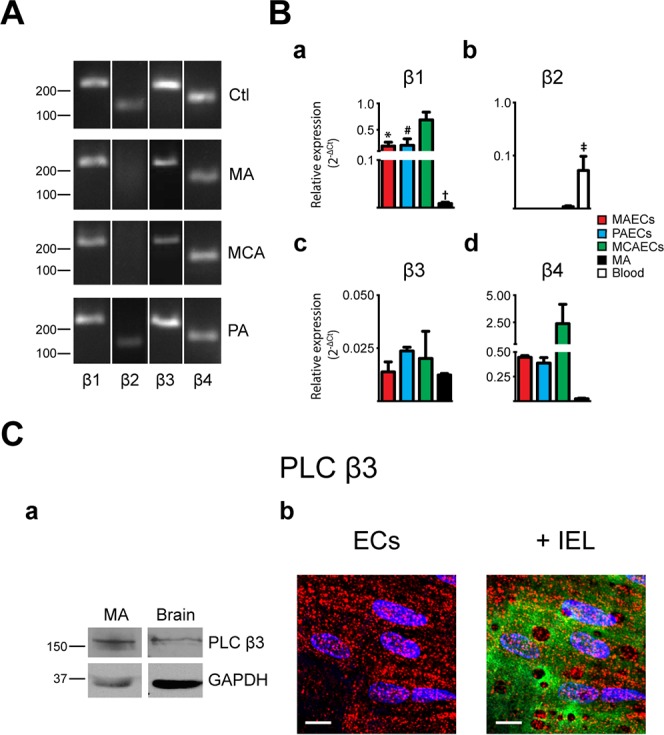
Characterization of phospholipase C β isoforms in native arteries. **A.** The presence of mRNA for phospholipase C (PLC) β isoforms was determined in mesenteric arteries (MA), pulmonary arteries (PA) and middle cerebral arteries (MCA) by PCR. Typical agarose gel electrophoresis of the PCR products showed the expression profile in different vascular beds. Brain and blood were used as positive control tissues. n = 3. **B.** Quantitative real time PCR analysis of mRNA expression levels of PLCβ isoforms in MA and freshly isolated endothelial cells (ECs) from MA, PA and MCA. Bar graphs show the expression profile of PLCβ1 (a), β2 (b), β3 (c) and β4 (d) isoforms in MAECs, PAECs, MCAECs, MA and blood as control for β2. n = 3. * P<0.05 between MAECs and MCAECs; # P<0.05 between PAECs and MCAECs; † P<0.05 between MCAECs and MAs; ‡ P<0.05 between control tissue and MA. **C.** (a) Representative immunoblots of murine MA and brain that were obtained using the primary antibody anti-PLC β3 (Abcam #ab52199). GAPDH was used as reference protein. Relevant molecular weight markers are indicated on the left. n = 3. (b) Intracellular distribution of PLCβ3 immunoreactivity in ECs. (Left) Typical image showing labelling of PLC β3 in red and nuclei in blue; scale = 10 μm. (Right) Labelling of PLC β3 (red) overlay with internal elastic lamina (IEL; green) where voids correspond to potential myoendothelial projections; nucleus in blue; scale = 10 μm; n = 4.

**Fig 2:**
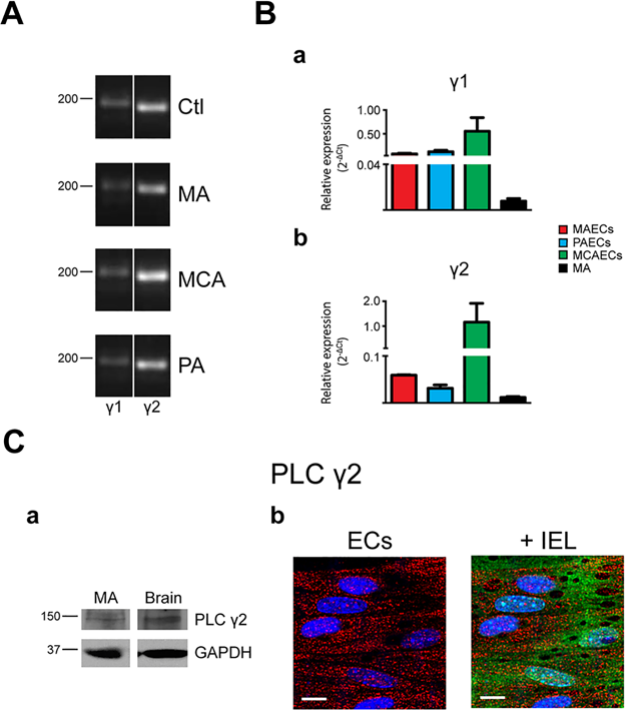
Characterization of phospholipase C γ isoforms in native arteries. **A.** The presence or mRNA for phospholipase C (PLC) γ isoforms was determined in mesenteric arteries (MA), pulmonary arteries (PA) and middle cerebral arteries (MCA) by PCR. Typical agarose gel electrophoresis of the PCR products showed the expression profile in the different vascular beds and brain was used as control tissue. n = 3. **B.** Quantitative real time PCR analysis of mRNA expression levels of PLCγ isoforms in MA and freshly isolated endothelial cells (ECs) from MA, PA and MCA. Bar graphs show the expression profile of PLCγ1 (a) and γ2 (b) isoforms in MAECs, PAECs, MCAECs and MA. n = 3. **C.** (a) Representative immunoblots of murine MA and brain that were analyzed using the primary antibody anti-PLC γ2 (Abcam #ab18983). GAPDH was used as reference protein. Relevant molecular weight markers are indicated on the left. n = 3. (b) Intracellular distribution of PLCγ2 immunoreactivity in ECs. (Left) Typical image showing labelling of PLCγ2 in red and nuclei in blue; scale = 10 μm. (Right) Labelling of PLCγ2 (red) overlay with internal elastic lamina (IEL; green) where voids correspond to potential myoendothelial projections; nucleus in blue; scale = 10 μm; n = 4.

**Fig 3:**
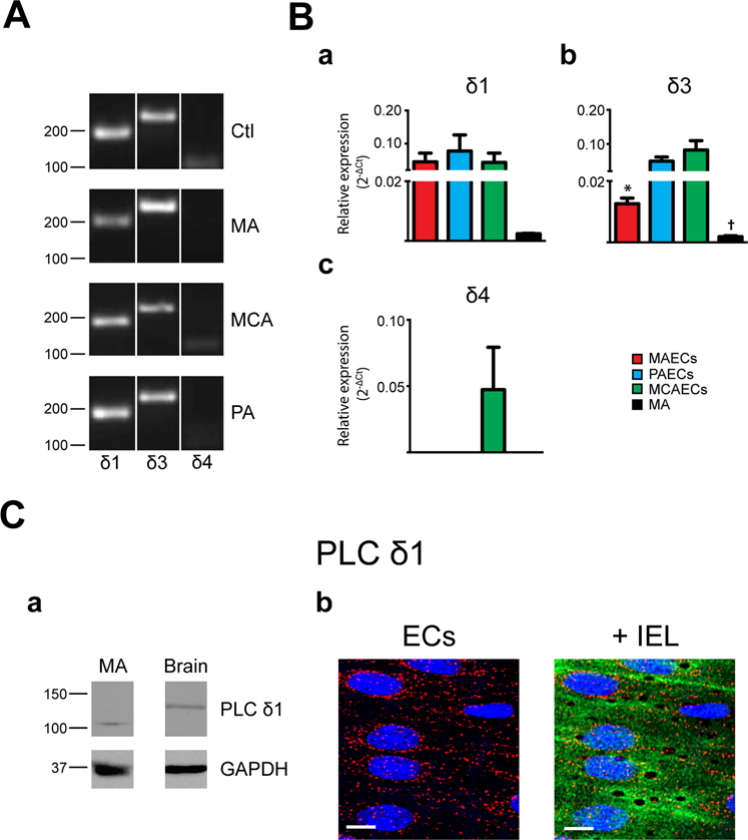
Characterization of phospholipase C δ isoforms in native arteries. **A.** The presence of mRNA for phospholipase C (PLC) δ isoforms was determined in mesenteric arteries (MA), pulmonary arteries (PA) and middle cerebral arteries (MCA) by PCR. Typical agarose gel electrophoresis of the PCR products showed the expression profile of PLCδ isoforms in the different vascular beds and brain was used as control tissue. n = 3. **B.** Quantitative real time PCR analysis of mRNA expression levels of PLCδ isoforms in MA and freshly isolated endothelial cells (ECs) from MA, PA and MCA. Bar graphs showed the expression profile of PLCδ1 (a), δ3 (b) and δ4 (c) isoforms in MAECs, PAECs, MCAECs and MA. n = 3. * P<0.05 between MAECs and MCAECs; † P<0.05 between MCAECs and MA. **C.** (a) Representative immunoblots of murine MA and brain that were analyzed using the primary antibody anti-PLCδ1 (Abcam #ab154610). GAPDH was used as reference protein. Relevant molecular weight markers are indicated on the left. n = 3. (b) Intracellular distribution of PLCδ1 immunoreactivity in ECs. (Left) Typical image showing labelling of PLCδ1 in red and nuclei in blue; scale = 10 μm. (Right) Labelling of PLCδ1 (red) overlay with internal elastic lamina (IEL; green) where voids correspond to potential myoendothelial projections; nucleus in blue; scale = 10 μm; n = 4.

**Fig 4:**
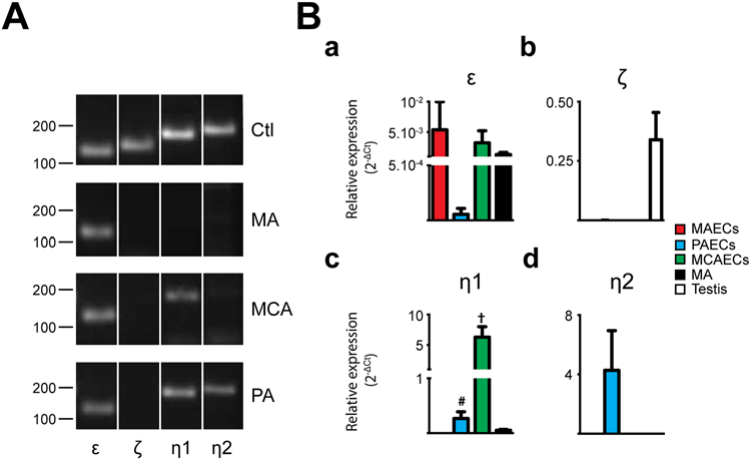
Characterization of phospholipase C ε, ζ and η isoforms in native arteries. **A.** The presence of mRNA for phospholipase C (PLC) ε, ζ, η1 and η2 isoforms was determined in mesenteric arteries (MA), pulmonary arteries (PA) and middle cerebral arteries (MCA) by PCR. Typical agarose gel electrophoresis of the PCR products showed the expression profile of PLCε, ζ, η1 and η2 isoforms in the different vascular beds and brain or testis were used as control tissue. n = 3. **B.** Quantitative real time PCR analysis of mRNA expression levels of PLCε, ζ, η1 and η2 isoforms in MA and freshly isolated endothelial cells (ECs) from MA, PA and MCA. Bar graphs show the expression of PLCε (a), ζ (b), η1 (c) and η2 (d) isoforms in MAECs, PAECs, MCAECs, MA and testis as positive control for ζ. n = 3. * P<0.05 between MAECs and MCAECs; # P<0.05 between PAECs and MCAECs; † P<0.05 between MCAECs and MA.

Expression of PLC isoforms was initially determined in freshly isolated arteries from 3 different vascular beds: MA, PA and MCA (Figs 1–4, panels A). While mRNA for 8 of the 13 PLC isoforms were detected in MA, 10 were found in MCA and 11 in PA. As shown in Fig 1A, PLCβ1, β3 and β4 mRNA was detected in all vascular beds studied. Interestingly, PLCβ2 was only detected in PA. PLCγ and ε isoforms were expressed in all arteries tested (Figs 2A and 4A). While PLCδ1 and δ3 were in all tested arteries, only MCA express δ4 (Fig 3A). PLC ζ was only detected in testis, our positive control, with no noticeable expression in arteries (Fig 4A). The PLCη family had a distinct expression pattern, as shown in Fig 4A. PLCη1 was found in MCA and PA but not in MA, whilst η2 was detected only in PA. Data from PCR experiments are summarized in Table 2. However, these expression profiles represent mRNA from all cells found in the arterial wall, including both endothelium and vascular SMCs. Assessment of endothelial-specific PLC expression requires isolation of mRNA from preparations of ECs.

**Table 2:** Summary of mRNA expression for phospholipase C isoforms.

	qPCR				PCR		
	MAECs	PAECs	MCAECs	MA	MA	PA	MCA
PLCβ1	+	+	+	+	+	+	+
PLCβ2	-	-	-	-	-	+	-
PLCβ3	+	+	+	+	+	+	+
PLCβ4	+	+	+	+	+	+	+
PLCγ1	+	+	+	+	+	+	+
PLCγ2	+	+	+	+	+	+	+
PLCδ1	+	+	+	+	+	+	+
PLCδ3	+	+	+	+	+	+	+
PLCδ4	-	-	+	-	-	-	+
PLCε	+	+	+	+	+	+	+
PLCζ	-	-	-	-	-	-	-
PLCη1	-	+	+	+	-	+	+
PLCη2	-	+	-	-	-	+	-

*MAECs*, *mesenteric arteries endothelial cells; PAECs*, *pulmonary arteries endothelial cells; MCAECs*, *middle cerebral arteries endothelial cells; MA*, *mesenteric arteries; PA*, *pulmonary arteries; MCA*, *middle cerebral arteries*.

Endothelial PLC expression was quantified in freshly isolated MAECs, PAECs and MCAECs by qPCR. The purity of EC isolations was assessed by determining the expression ratio of EC *vs*. SMC markers (CD31 and SM22, respectively; S1 Fig) in both isolated arteries and corresponding EC preparations. This assessment confirmed that EC samples used were highly enriched in ECs. The relative expression of PLC isoforms in MAECs, PAECs and MCAECs as well as in MA was then determined (Figs 1–4, panels B). PCR and qPCR yielded similar expression profiles for PLC mRNA in MA with the exception of PLCβ2 and η1, which were only detected using the more sensitive qPCR. Therefore, mRNA encoding for nine PLC isoforms was detected in MA by qPCR. An almost identical PLC profile was observed in MAECs, with the exception that PLCη2 was undetectable in MAECs (Fig 4B). From a vascular bed point of view, slight differences were found between MAECs, PAECs and MCAECs. ECs from all arteries tested expressed at least 8 of the 13 PLC isoforms: β(1,3,4), γ(1–2), δ(1,3) and ε. Additionally, PAECs and MCAECs expressed PLCη1, whereas MAECs did not. In accordance with our findings in whole arterial preparations, PLC δ4 mRNA was only detected in MCAECs and PLCη2 was only detected in PAECs. These results are summarized in Table 2.

### Protein expression of PLC isoforms

The three main PLC families are PLCβ, PLCγ and PLCδ [26]. However, investigation of PLC isoform expression at the protein level was limited to the commercially available antibodies: PLCβ3, PLCγ2 and PLCδ1. Western blotting was thus used to elucidate the expression of these PLC isoforms in MA at the protein level. As shown in Fig 1Ca, PLCβ3 antibodies revealed a band of 150-kDa in MA extracts. PLCδ1 and PLCγ2 antibodies revealed bands of 100-kDa and 150-kDa, respectively (Figs 2Ca and 3Ca).

### Subcellular distribution of PLC isoforms

Since PLCβ3, γ2 and δ1 were detected in MA by western blotting, we next determined their respective presence and subcellular distribution. Immunofluorescence experiments were performed on MA sections dissected and immobilized with the endothelium in an *en face* configuration. All isoforms displayed a similar intracellular distribution. PLCβ3 (Fig 1Cb), γ2 (Fig 2Cb) and δ1 (Fig 3Cb) were homogeneously distributed in intact endothelium of MA.

## Discussion

PLCs are crucial components of the phosphoinositide signalling pathway through their hydrolysis of PIP_2_ into IP_3_ and 1,2-DAG. The 13 mammalian PLC isozymes identified so far are organized within 6 families: β(1–4), γ(1–2), δ(1,3,4), ε, ζ and η(1–2) [5]. PLC isoforms are distinct in their activation mode, expression levels, catalytic regulation, cellular localization and tissue distribution. PLCβ and γ are considered as “first line PLCs” as they are activated by extracellular stimuli. In contrast, PLCδ, ε, η and ζ are secondary PLCs, being activated by intracellular Ca^2+^ [27]. For example, PLCγ isoforms are activated by tyrosine kinase receptors while members of the PLCβ family are activated by G protein-coupled receptors [28,29]. These distinct characteristics allow PLC activity to be involved in a wide range of functions in both physiology and pathophysiology [30]. For example, each neuronal PLC isoform selectively couples with a specific neurotransmitter and contributes to distinct functions. PLCβ1 knockout mice are afflicted with epilepsy while abnormal activity and expression level of PLCγ1 are detected in pathologies including Huntington's disease, depression and Alzheimer's disease [7,31]. These numerous pathophysiological roles illustrate the extensive variety of cellular functions associated with each individual PLC isoform. Despite their crucial role in cellular signalling, the expression pattern of PLC isozymes remains to be established in native ECs. This study is the first study of PLC isoform expression at the mRNA level in murine resistance arteries from 3 distinct vascular beds: MA, PA and MCA. We also examined PLC expression pattern in freshly isolated ECs from these arterial beds. Finally, we demonstrated the presence of immunoreactivity for 3 major PLC isoforms (β3, γ2 and δ1) in MA and their homogenous subcellular distribution in MA endothelium.

Recently, Lo Vasco and coll. Report that of 10out of 13 PLC isoforms are expressed in HUVECs, a cultured ECs model [18]. Interestingly, our data slightly differs from that of Lo Vasco *et al*.: PLCβ1 and ε are expressed in native ECs from all vascular beds tested but not in HUVECs. However, β2 is absent from freshly isolated murine ECs but is expressed in HUVECs. Also, η1 and η2 are expressed in HUVECs but were not detected in the 3 types of native ECs. These discrepancies might be explained by the alteration of endothelial phenotype maintained in culture [32]. In vitro, ECs lose their MEP, an essential architecture for the communication with vascular SMCs. Therefore, in addition to passaging, culture media and the absence of shear stress, the loss of cellular polarity may contribute to the observed differences in PLC expression in native and cultured ECs. Moreover, while we used arterial tissues in the present study, Lo Vasco and coll. used cells from veins. Hence, arteries to veins differences may also explain the different expression reported.

Employing qPCR allowed us to identify the PLC isoforms expressed specifically in freshly isolated ECs from MA, PA and MCA. Three of the four known PLCβ isoforms were detected in all ECs tested: PLCβ1, β3 and β4. Moreover, PLCβ3 expression was also found at the protein level with immunoblots and confocal imaging. This is an interesting finding since PLC β3 is involved in the VEGF-dependent inhibition of vascular permeability [33]. However, the roles of endothelial β1 and β4 remain to be determined. Undetectable expression of PLC β2 from ECs was anticipated considering its involvement in platelets and hematopoietic cells chemotaxis [5,34–36]. Both PLCγ isoforms were detected in native ECs. PLCγ1 appears to be required for normal development as knockout mice do not survive beyond E9, due to a generalized growth failure attributed to the loss of both erythroid progenitors cells and ECs necessary for erythropoiesis and vasculogenesis [37,38]. On the other hand, PLCγ2 appears to be selectively expressed in blood cells, spleen and thymus [39]. The present study is the first report of PLCγ2 expression in native ECs. Detection of PLCγ2 expression by qPCR was further supported by immunoblotting. Moreover, immunostaining showed a homogeneous endothelial distribution of the enzyme. Obviously, the specific role of PLCγ2 in ECs is yet to be elucidated. Consistent with reports of PLCδ subtypes being expressed in porcine aortic ECs [40], we detected all three PLCδ isoforms in MCAECs whilst PLCδ4 was absent from MAECs and PAECs. PLCδ isoforms are among the most sensitive to Ca^2+^, suggesting a potential role downstream of changes in endothelial Ca^2+^ dynamics [41,42]. However, elucidating the role of PLCδ4 specific function in MCAECs will require additional investigation. Studies employing a PLCδ4-null mouse have shown PLCδ4 to be involved in the initial stages of fertilization [6] but its role in endothelial function remains to be established. Immunoblotting of PLC δ1 revealed differences in apparent molecular weight in MA and brain. This can be explained by various types of post-translational modification, such as multiple phosphorylations as described by Fujii *et al*. [43]. Finally, we detected PLCε, but not PLCζ, in freshly isolated ECs from all vascular beds. In contrast, while PLCη2 is only expressed in PAECs, PLCη1 was not detected in MAECs. Similar to δ4, there is currently no information available regarding the expression or function of PLCε, ζ, η1 and η2 in ECs. However, the function of these PLC isoforms has been examined in other cell types. For example, PLCε-null mice show abnormal development of the aortic and pulmonary valves [9], while PLCζ expression/function was reported as sperm-specific [44] and PLCη1 and η2 seem to be involved in neural system regulation [5]. In summary, very little is known about the function of PLC isoforms in ECs, and further studies are therefore required to investigate their respective and specific roles.

ECs are heterogeneous both in structure and function across the vascular tree under normal or pathological conditions [20,21]. For example, rat PAECs have a broader and shorter shape than rat aortic ECs [45]. ECs are involved in several physiological functions, the relative importance of which varies according to blood vessel type (conduit *vs*. resistance) or vascular bed, including vascular permeability, hemostasis or vasomotor tone in response to the specific requirements of the perfused organ. For example, pulmonary vasculature is exposed to a low-pressure, high flow of oxygen-deprived blood. Therefore, the proteins expressed in ECs will have varied in order to adapt to the needs of their particular vascular bed of origin [19]. We determined the expression of all known mammalian PLC isoforms in native ECs of resistance arteries from three different vascular beds (MA, PA and MCA) by qPCR and observed a similar pattern of expression in each case. For 10 isoforms, the pattern of expression was constant between the 3 vascular beds (8 isoforms detected while 2 other were not detected). However, we observed slight differences for 3 isoforms: PLCδ4 and η2 were detected exclusively in MCAECs and PAECs, respectively, whereas PLCη1 was expressed in MCAECs and PAECs but not MAECs.

Endothelial PLC might be involved in the modulation of Ca^2+^ dependant vasoregulatory signals. In fact, the release of Ca^2+^ through IP_3_ receptors in the ER membrane is stimulated by the IP_3_ generated upon activation of membrane receptors leading to activation of PLC. PLC are known to regulate Ca^2+^ levels and thus might be involved in the modulation of intracellular Ca^2+^ dynamics. In 2013, De Bock and coll. reviewed the signalling pathways including PLCs pathway leading to intracellular Ca^2+^ elevation in blood-brain-barrier ECs [46]. Although all PLCs are Ca^2+^-dependent, they vary with respect to their sensitivity to Ca^2+^. PLCδ and η are the most sensitive to Ca^2+^ and are involved in potentiating Ca^2+^ signalling [47,48]. In a recent study, PLCβ1 and β4 were shown to be involved in distinct histamine-induced Ca^2+^ oscillations [49].

In ECs, increased IP_3_ production results in the release of Ca^2+^ from intracellular stores [50] and the IP_3_R is the only ER Ca^2+^ channels expressed in these cells [51]. IP_3_ is generated through activation of G protein-coupled receptors or tyrosine kinase receptors which activate PLCβ and γ respectively [52]. Multiple cellular functions are regulated by changes in intracellular Ca^2+^. Therefore, specificity of the Ca^2+^ signal is often achieved through spatial and temporal compartmentalization of Ca^2+^ signals. Several different patterns of Ca^2+^ dynamics have recently been described in ECs: Ca^2+^ pulsars, TRPV4- and TRPA1-sparklets or wavelets, each generated by a specific pathway [11,12,14,53]. Ca^2+^ pulsars are MEP localized signals consisting of a spontaneous Ca^2+^ release from IP_3_ receptors [11]. Limited information is currently available on the regulatory mechanisms of Ca^2+^ pulsars. However, specific PLC activation could be responsible for localized Ca^2+^ signalling such as Ca^2+^ pulsars. Cytoplasmic gradients of IP_3_ would result in a limited activation of IP_3_R and restricted propagation of the signal. Although our immunochemistry data showed a homogeneous distribution of PLCβ3, γ2 and δ1 in ECs, PLC partners and modulators could be heterogeneously distributed within the cell. Microdomains would then result from these associations as well as the distribution of proteins involved in Ca^2+^ sequestration. For example, Gonzales and coll. have recently shown colocalization of PLCγ1 with IP_3_R, TRPM4 and TRPC6 in VSMC from cerebral arteries and elegantly demonstrated its requirement for pressure-induced membrane depolarization and myogenic vasoconstriction [13]. Moreover, TRPV4 channels responsible for Ca^2+^ sparklets are clustered in EC microdomains at MEP where they are modulated by PLC isoforms [54,55]. As for TRP channels, specific PLC isoforms can be localized in microdomains to have a pivotal role in the modulation of Ca^2+^ pulsars [56]. Moreover, our investigation of the subcellular localization of PLC was limited to three isoforms due to a lack of commercially available, reliable antibodies for the other isoforms. Therefore, the subcellular distribution of other members of the PLC family in native endothelium remains to be determined.

In summary, in this study we established for the PLCs that are expressed in native endothelium and freshly isolated ECs. We showed that 8 out of the 13 mammalian PLC isoforms are expressed in MAECs and 10 in PAECs and MCAECs. These results represent an important step forward in our understanding of the intracellular signalling pathways and their role in the regulation of endothelial microdomains. Further investigation of the subcellular distribution and biological function of these PLC isoforms is required in order to have a better understanding of their relative involvement in regulating endothelial function.

## Supporting Information

S1 Fig Purity of freshly isolated endothelial cells samples. Quantitative real time PCR analysis of mRNA expression levels of CD31, an endothelial-specific marker, and SM22, a smooth muscle cell-specific marker. (A) Pie chart illustrating the relative expression of CD31 to SM22 (CD31/SM22 ratio) in mesenteric arteries (MA) and in endothelial cells isolated from mesenteric arteries (MAECs). (B) Pie chart illustrating CD31/SM22 ratio in pulmonary arteries (PA) and in endothelial cells from pulmonary arteries (PAECs). (C) Pie chart illustrating CD31/SM22 in middle cerebral arteries (MCA) and in endothelial cells from middle cerebral arteries (MCAECs). n = 3.
(TIF)
