# Supplementary figures and images for: Expression of Phosphoinositide-Specific Phospholipase C Isoforms in Native Endothelial Cells
Source: PLoS One. 2015 Apr 13;10(4):e0123769. doi: 10.1371/journal.pone.0123769 (PMC4395365; doi:10.1371/journal.pone.0123769)

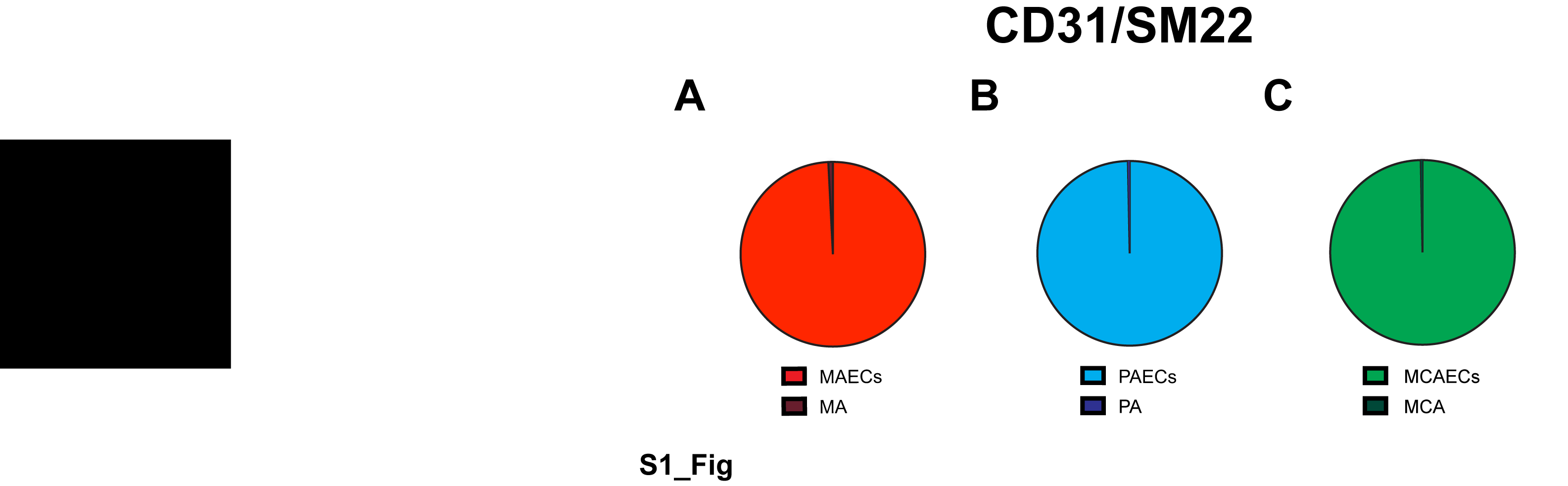

Supplement: S1 Fig — Quantitative real time PCR analysis of mRNA expression levels of CD31, an endothelial-specific marker, and SM22, a smooth muscle cell-specific marker. (A) Pie chart illustrating the relative expression of CD31 to SM22 (CD31/SM22 ratio) in mesenteric arteries (MA) and in endothelial cells isolated from mesenteric arteries (MAECs). (B) Pie chart illustrating CD31/SM22 ratio in pulmonary arteries (PA) and in endothelial cells from pulmonary arteries (PAECs). (C) Pie chart illustrating CD31/SM22 in middle cerebral arteries (MCA) and in endothelial cells from middle cerebral arteries (MCAECs). n = 3. (TIF) [file pone.0123769.s001.tif]
